# Whole-Exome Sequencing Reveals Pathogenic SIRT1 Variant in Brain Arteriovenous Malformation: A Case Report

**DOI:** 10.3390/genes13101689

**Published:** 2022-09-21

**Authors:** Kymbat Mukhtarova, Elena Zholdybayeva, Ulykbek Kairov, Ilyas Akhmetollayev, Chingiz Nurimanov, Marat Kulmirzayev, Yerbol Makhambetov, Yerlan Ramankulov

**Affiliations:** 1 National Center for Biotechnology, 13/5 Kurgalzhynskoye Road, Astana 010000, Kazakhstan; 2 Laboratory of Bioinformatics and Systems Biology, Center for Life Science, National Laboratory Astana, Nazarbayev University, 53 Kabanbay Batyr Ave., Astana 010000, Kazakhstan; 3 National Center of Neurosurgery, 34/1 Turan Street, Astana 010000, Kazakhstan; 4 School of Science and Technology, Nazarbayev University, 53 Kabanbay Batyr Ave., Astana 010000, Kazakhstan

**Keywords:** brain arteriovenous malformation, whole exome sequencing, case study, SNP, somatic mutations

## Abstract

Arteriovenous malformations of the brain (bAVMs) are plexuses of pathological arteries and veins that lack a normal capillary system between them. Intracranial hemorrhage (hemorrhagic stroke) is the most frequent clinical manifestation of AVM, leading to lethal outcomes that are especially high among children and young people. Recently, high-throughput genome sequencing methods have made a notable contribution to the research progress in this subject. In particular, whole-exome sequencing (WES) methods allow the identification of novel mutations. However, the genetic mechanism causing AVM is still unclear. Therefore, the aim of this study was to investigate the potential genetic mechanism underlying AVM. We analyzed the WES data of blood and tissue samples of a 30-year-old Central Asian male diagnosed with AVM. We identified 54 polymorphisms in 43 genes. After *in-silica* overrepresentation enrichment analysis of the polymorphisms, the *SIRT1* gene variant (g.67884831C>T) indicated a possible molecular mechanism of bAVM. Further studies are required to evaluate the functional impact of *SIRT1* g.67884831C>T, which may warrant further replication and biological investigations related to sporadic bAVM.

## 1. Introduction

Arteriovenous malformations (AVMs) of the brain (OMIM, #108010) consist of pathological arteries and veins that lack a normal capillary system between them. Brain arteriovenous malformations (bAVMs) are among the major causes of intracranial hemorrhage and epilepsy. Detection of AVMs early in childhood is considered more dangerous than identification in adulthood because the risk of AVM rupture is highest in the age group of 15 to 40 years [1].

Recent advances in genomic and other multi-omics technologies have provided insights into the etiology of AVMs. Previously, AVMs were entirely considered to be congenital lesions. Nevertheless, numerous studies have described the appearance of AVMs in the postembryonic period. Moreover, the properties of AVMs significantly change with time. Therefore, a novel theory about the *de novo* mechanism of AVM has emerged [2]. Currently, AVMs are divided into hereditary and sporadic forms. More than 95% of cases are sporadic, and approximately 3% of AVMs are caused by hereditary hemorrhagic telangiectasia (HHT), known as Osler-Weber-Rendu disease, in an autosomal dominant mode of inheritance [3]. Mutations in the *ENG* (OMIM:131195), *ACVRL1* (OMIM:601284), and *SMAD4* (OMIM:600993) genes are associated with HHT. All three genes encode proteins involved in the transforming growth factor TGF-β signaling pathway [4]. However, as noted in previous studies, there are other genes that cause HHT that have not yet been identified. These are presumably localized on chromosomes 5 and 7 [5,6]. The hereditary form of AVM also includes capillary malformations and arteriovenous malformations (CM-AVM, OMIM # 608354) caused by mutations in the *RASA1* gene [7]. In addition, recent literature suggests AVMs that have been considered stable for many years have a dynamic nature [8].

The pathogenesis of sporadic AVM remains poorly understood. Studies on candidate genes and genetic factors that contribute to the development of sporadic forms of AVM began in 2000. Weinsheimer et al. presented the results of the first genome-wide association study (GWAS) in European patients with sporadic bAVM [9]. Thomas et al., in their review, noted that the pathogenesis of the disease is unknown, and that markers of disease development were not identified. Therefore, they do not exclude the role of epigenetic factors along with genetic factors [10].

In 2018, Nikolaev et al. hypothesized that bAVMs could develop as a result of KRAS-induced activation of the MAPK-ERK signaling pathway in the brain endothelial cells. They found activating somatic mutations in *KRAS* in tissue samples with AVM. This was important for the development of targeted therapies for AVMs [11]. Consequently, several studies have been conducted in this regard. Hong et al. investigated the genetic profiles of AVMs of the brain and spinal cord in patients and found somatic tumor mutations. The prevalence of *KRAS/BRAF* mutations was 81.0% (17 out of 21) in the brain and 100% (10 out of 10) in spinal column AVMs. For the first time, the mechanisms of activating *BRAF* mutations and two new mutations in *KRAS* (p.G12A and p.S65_A66insDS) in the central nervous system with AVMs were detected. The study emphasized the importance of these results in the development of targeted bAVM treatment [12]. Walcott et al. investigated potential mutations in a 14-year-old girl who developed a recurrent bAVM using whole-exome sequencing of the AVM tissue lesions and blood. A stop–gain mutation was observed (c.C739T:p.R247X) in the *SMAD* family member 9 (*SMAD9*) [13].

Thus, based on the different genes identified that can contribute to AVMs, it is postulated that the pathogenesis of AVM is not fully understood and requires further investigations. Therefore, this study aimed to identify potential genetic risk factors for sporadic bAVMs in a young Central Asian adult using whole-exome sequencing techniques.

## 2. Materials and Methods

### 2.1. Data Collection

This study was approved by the ethics committee of the National Center for Biotechnology (#2/01.08.2019 (Nur-Sultan, Kazakhstan)) and was conducted according to the principles expressed in the Declaration of Helsinki. Informed consent was obtained from all participants.

Following the surgical removal of the bAVM from the 30-year-old man, DNA samples were extracted from the blood and tissue samples. The inclusion criteria were diagnosis of bAVM based on computed tomography (CT) or magnetic resonance angiography (MRA) data and confirmed at surgery, when applicable. AVM tissue specimens were collected during a clinically indicated procedure, flash-frozen, and stored at −80 °C until further use.

### 2.2. DNA Isolation

DNA was extracted from the frozen tissue samples and peripheral blood using a DNeasy Blood and Tissue Kit (Qiagen, Hilden, Germany). Genomic DNA was isolated from 9 mL of EDTA-anticoagulated whole venous blood using a standard salt-out method (Miller et al. 1988). DNA concentrations were determined by measuring absorbance at 260 nm using a Nanodrop spectrophotometer (Thermo Fisher Scientific, Waltham, MA, USA).

### 2.3. Whole Exome Sequencing (WES)

WES was performed on the bAVM tissue and blood samples to identify genetic variants contributing to sporadic bAVM. WES was performed at Macrogen (Seoul, Korea).

Genomic DNA libraries were prepared according to the standard protocol provided by Illumina, Inc. The protein-coding regions of human genomic DNA were identified using an Agilent SureSelect V6-Post kit (Agilent, Santa Clara, CA, USA) and sequenced on a Novaseq 6000 platform (Illumina Inc., San Diego, CA, USA) (Table 1).

### 2.4. WES Data Processing

Single-nucleotide variants (SNVs) and insertion/deletions (indels) were detected using the Genome Analysis Toolkit according to GATK best practice guidelines. Annotation of genetic variants (ANNOVAR) tools were used to annotate the variants for location, corresponding genes, and transcript length.

The GATK workflow was used for the analysis and annotation of the raw data, and vcf files were generated. Then, the variants were filtered for a quality ≥ 20 in Excel. The resulting variants obtained were analyzed for protein coding/exonic regions, resulting in identification of amino acid change/nonsynonymous, missense, stop–lost, stop–gained, and frameshift insertion deletions.

### 2.5. Gene Ontology (GO), Disease and Pathway Over-Representation Analysis (ORA)

To investigate the main functional mechanisms of the genes with mutations in the blood and tissue samples, the GO analysis was divided into cellular components, molecular functions, biological processes, and the KEGG. Pathway enrichment analysis of shortlisted genes and variants was performed by Overrepresentation Enrichment Analysis (ORA) via WebGestalt (WEB-based GEne SeT AnaLysis Toolkit) (http://www.webgestalt.org, accessed on 16 January 2022).

### 2.6. Mutation Validation

Potential pathogenic variants detected by WES were confirmed by DNA Sanger sequencing. Polymerase chain reaction (PCR) primers were designed, as shown in Table 2.

Amplified PCR was performed in a final volume of 20 µL, containing 50 ng/µL of the genomic DNA, 10 pmol of the forward and reverse primers (SIRT_F and SIRT_R), 3% DMSO, 0.4 U of Phusion™ High-Fidelity DNA Polymerase (Thermo Fisher, Cleveland, OH, USA), 2.5 mM of each dNTP (Fermentas), and 4 µL of 5× PCR buffer. The thermal cycling conditions included initial denaturation for 30 s at 98 °C, followed by 10 cycles at 98 °C for 10 s, 72 °C (−1.6 °C/cycle) for 20 s, and 72 °C for 1 min and 30 s. Then, 30 cycles of denaturation at 98 °C for 10 s, annealing at 64 °C for 20 s, elongation at 72 °C for 1 min and 30 s, and a final elongation for 5 min at 72 °C. The PCR products were run on a 1% agarose gel and were then purified using a QIAquick PCR purification kit (Qiagen), according to the standard manufacturer’s protocol. The extracted PCR product was digested using Exo-Sap enzymes (Thermo Fisher Scientific, Wilmington, Germany). DNA sequencing was performed on the digested PCR products using the BigDye Terminator Cycle Sequencing v.3.1 kit (Applied Biosystems, Foster City, CA, USA). DNA sequencing analysis was performed using an ABI 3730XL Genetic Analyzer (Applied Biosystems, Foster City, CA, USA).

## 3. Results

### 3.1. Case Presentation and Surgical Procedure

**Figure 1:**
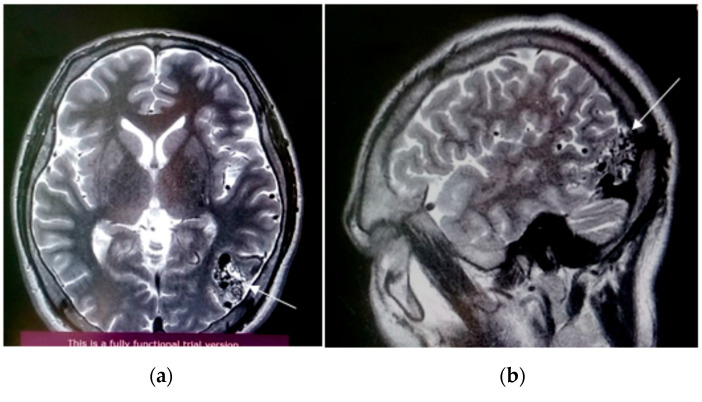
Axial (**a**) and sagittal (**b**) views of MRI obtained at initial presentation of the patient. AVM in the middle cerebral artery (MCA) and hypoplasia of cerebellar tonsils with subarachnoid hemorrhage (SAH). Right sided sinusitis and ethmoiditis. White arrows indicate location of AVM on the MRI.

A 30-year-old Central Asian male presented to the clinic with an AVM of 3–6 mm in the left occipital lobe with a Spetzler-Martin grade II. Furthermore, the surface venous drainage had no ruptures. The patient reported no family history of hereditary disease. The patient confirmed that his closest relatives, who had episodes of intracranial hemorrhage, were not diagnosed with AVM. The major symptoms were headache and convulsive seizures of a tonic–clonic nature, with impaired consciousness with a duration of 5–10 min and at a frequency of 3–4 times monthly. From the patient’s history, he had been experiencing headaches for several years, and he associated this pain with a head trauma he suffered in 2012. His condition worsened in October 2019, when the pain was accompanied by convulsive seizures and loss of consciousness. The patient consulted a neurologist and magnetic resonance imaging (MRI) of the brain was performed (Figure 1). The patient was consulted by a neurosurgeon, and surgical treatment was recommended. Consequently, the patient was hospitalized in September 2020. Super-selective cerebral angiography was performed. X-ray endovascular partial embolization of an AVM in the left occipital lobe was performed using Phil-25. No postoperative complications were reported.

Following discharge, the patient experienced convulsive seizures. Antiepileptic drugs were not prescribed. At the time of sample collection, the patient was hospitalized for the second stage of the surgical treatment. First, partial embolization of the superior posterior part of the AVM in the left temporal lobe was performed, preserving afferents from the temporal branch of the left middle cerebral artery and the left posterior cerebral artery. Moreover, efferent veins through the Labbe vein into the left transverse sinus were preserved. Second, after one week, craniotomy of the left temporal-occipital region was performed via microsurgery to remove the AVM in the left temporal-occipital lobe. There were no complications in the postoperative period.

### 3.2. WES Results Analysis

The annotated results were analyzed for the presence of single-nucleotide polymorphisms (SNPs) and insertion-deletion mutations (Indels) between DNAs from the AVM tissue sample and blood. The variants were filtered for the quality of reads (52,794 variants) and genotype differences between tissue and blood DNA samples (2991 variants). After selecting only non-synonymous, frameshift insertions and deletion variants with different genotypes in the blood and tissue samples, 30 variations were observed in 23 genes (Table 3).

### 3.3. Over-Representation Analysis

**Figure 2:**
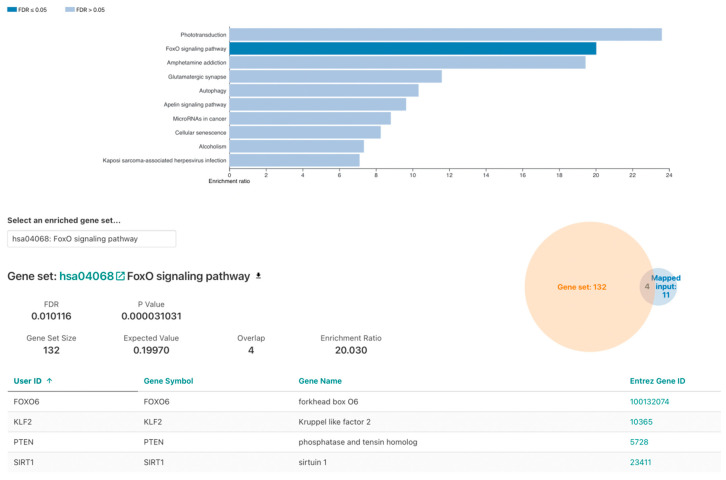
WebGestalt KEGG pathway ORA: FOXO signaling pathway.

ORA was conducted on 23 genes (Table 3). Significant functional enrichment (FDR < 0.05) was identified in the GO analysis muscle cell apoptotic process (Table S1). KEGG pathway analysis revealed significantly over-represented FOXO signaling pathway. Namely four out of the twenty-three analyzed genes, *SIRT1*, *PTEN*, *FOXO6* and *KLF2*, with *p* = 0.000031031 and FDR = 0.010116 (Figure 2) were identified. Regarding disease ORA, none of the findings were statistically significant or relevant (Table S1).

### 3.4. FOXO Signaling Pathway

**Figure 3:**
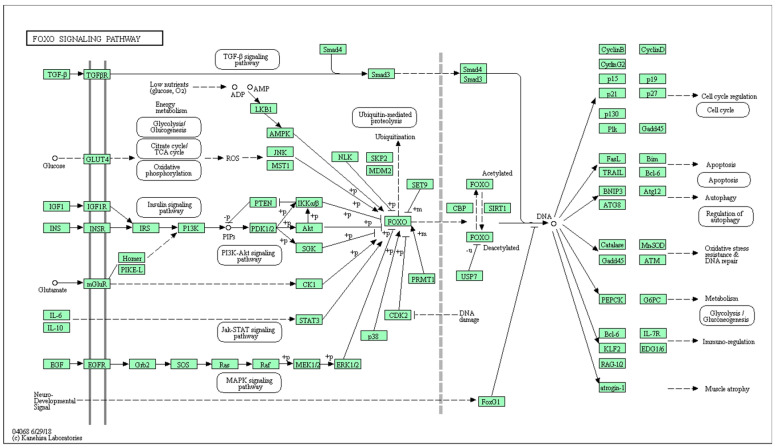
FOXO signaling pathway.

Overall, 130 genes were identified in the KEGG FOXO signaling pathway (hsa04068) (Figure 3).

*KLF2* is located in the downstream region of the FOXO signaling pathway, implying that it cannot control the FOXO signaling pathway. *PTEN* was not identified in WES conducted on the blood samples. However, *SIRT1* is located upstream in the signaling pathway and can modulate the pathway by controlling FOXO deacetylation.

**Figure 4:**
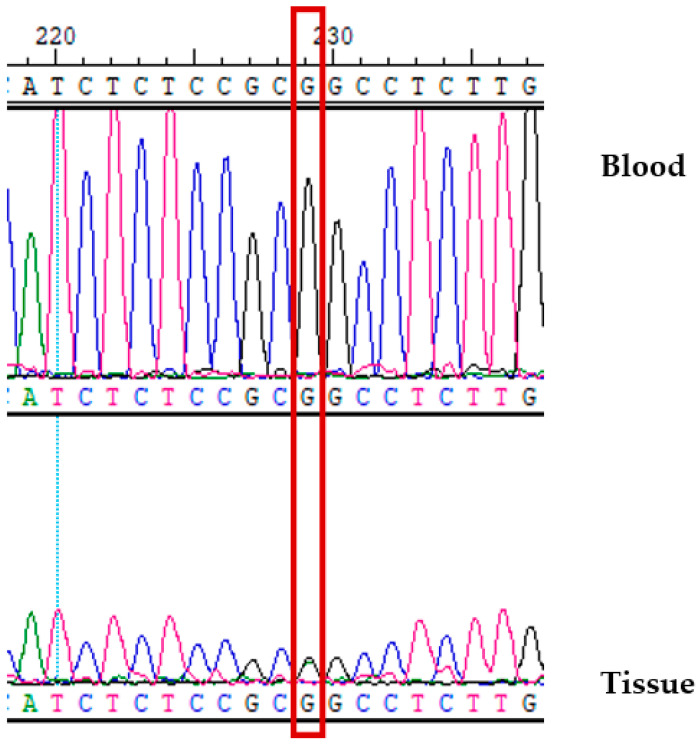
Validation analysis of the candidate variant. Sequence of *SIRT* in the affected patient’s blood and tissue. Here blue color indicates “C” allele, black—“G”, green—“A” and pink is for “T” allele.

The candidate genetic variant was sequenced by Sanger sequencing. The missense variant g.67884831C>T in SIRT was confirmed to be wild-type (CC) in the blood and heterozygote (CT) in the tissue (Figure 4). The variant was validated in 15 healthy individuals to eliminate any false–positive findings. MRI was performed for these individuals to exclude bAVM.

Within the exome aggregation consortium database, the minor allele frequency was reported to be 0.00077760. The score predictor of g.67884831C>T substitution pathogenicity was 0.003 for the Poly-Phen2, and this mutation was predicted as “benign”, but “damaging” for SIFT_pred (SIFT score −0.005) and for MutationTaster_pred (MutationTaster_score −0.997). The variant g.67884831C>T is a nonsynonymous substitution that leads to Pro37Leu substitution and is one of the frequent mutations at the N-terminus of SIRT1. In this case, we considered the rs548590752 mutation in SIRT to be somatic.

## 4. Discussion

An AVM is a fast-flow vascular anomaly consisting of connections between arteries and veins through a nidus or fistula instead of a normal capillary bed. Currently, there are many studies on somatic mutations associated with bAVMs [11,12,13].

In this study, WES was used to analyze the genetic profile of a patient with sporadic bAVM. Following processing of the sequencing results, only *SIRT* g.67884831C>T was a candidate variant.

Sirtuins (SIRTs, silent information regulators) are a family of evolutionarily conserved NAD-dependent proteins that possess deacetylase or ADP-ribosyltransferase activities [14]. To date, seven SIRTS have been identified, and they each have specific intracellular localization, functions, and substrate specificity [14].

SIRT1, SIRT6 and SIRT7 are nuclear proteins with distinct subcellular compartments. SIRT3, SIRT4, and SIRT5 are localized in the mitochondria, whereas SIRT2 is cytoplasmic. SIRTs1-3 have a strong deacetylase activity, while SIRTs4-7 have a weak deacetylase activity or no activity. SIRT4 has predominantly ADP-ribosyl transferase activity [15]. SIRT1 plays diverse roles in regulating cell proliferation, differentiation, stress responses, metabolism, energy homeostasis, aging, and cancer (Michishita et al. 2003, Cheng et al. [15,16,17,18]. Fulco et al. [18]). The sirtuin proteins contain a central region of high sequence conservation that is required for catalytic activity, but more variable N- and C-terminal regions have been proposed to mediate protein specific activities. N- and C-terminal segments of SIRT1 play autoregulatory functions to modulate SIRT1 deacetylase activity. Two of the most frequent genetic variations in the N terminus (S14P and P37L) increase SIRT1 protein activity [19].

Based on the results of this study, it would be interesting to consider the FOXO signaling pathway in AVM pathogenesis (Figure 2). FOXO is deacetylated by sirtuin-1 (SIRT1). Based on the studies described above, it is possible to assume that the presence of the P37L mutation in the amino acid sequence of the SIRT1 protein increases its activity, which, consequently, affects the deacetylation rate of FOXO. Adel Abuzenadah and his colleagues [20] noted that considering the wide range of functions of FOXO1 its expression rate can play a vital role. SIRT1 plays a unique role in vascular protection by regulating various substrates, including forkhead box O1 (FOXO1) [18]. A literature review by Abeer Dagra, et al., 2022, emphasized the importance of the role of SIRT1. Namely, they stated that it is a protein that is involved in regulation of the vasculature through various mechanisms, but importantly through vascular smooth muscle structural and functional homeostasis [21].

Nevertheless, the mechanisms of the interaction between FOXO1 and SIRT1 remain unclear. Potente et al. have shown that SIRT1 is highly expressed in the vasculature during the growth of blood vessels; it controls the angiogenic activity of endothelial cells. Impaired SIRT1 expression in zebrafish and mice leads to abnormal blood vessel formation. Moreover, at least part of the regulation of endothelial angiogenesis by SIRT1 occurs through modulation of FOXO1 transcriptional activity [22]. Forkhead box “O” (FOXO) transcription factors and sirtuin deacetylases are emerging as key regulators of mammalian vascular development and disease. Recent studies have shown that the interaction between FOXO1 and SIRT1 affects angiogenesis and tumor development in cancer. Kim et al. reported that the forkhead transcription factor FOXO1 inhibits angiogenesis in gastric cancer by inhibiting SIRT1 [23].

In summary, this study reported a rare *SIRT* variant g.67884831C>T that has an association with bAVM. This is a novel finding. SIRT-regulated FOXO signaling pathway affects the formation of blood vessels. It could be suggested that the identified mutation in the *SIRT* gene in the patient tissue samples affected the formation of blood vessels (arteries and veins) in the brain, leading to the development of bAVM. Further research is needed to establish a link between the candidate gene mutation detected in *SIRT1* by WES and the observed clinical phenotype, by studying in silico models.

## Figures and Tables

**Table 1:** Capture method and coverage summary of exome data.

	AVM	Blood
Capture method	Agilent SureSelect Human All Exon V6	
Mean coverage		
% > 10×	99.0	98.8
% > 20×	97.0	96.3
% > 30×	93.6	92.1

**Table 2:** Primers: sequences and characteristics.

Primer	Sequence (5′–3′)	Ta (°C)	Amplicon Length (bp)
SIRT_F	GACCCGTAGTGTTGTGGTCT	64	558
SIRT_R	TCGTCTTCGTCGTACAAGTTGTC	64	

**Table 3:** Summary of selected polymorphisms.

N	Chr	Position	SNP	Gene	REF	ALT	Type	Blood	Tissue
1	chr1	41847882	rs373524987	FOXO6	CA	-	frameshift deletion	0/1	1/1
2	chr1	41847886		FOXO6	C	G	nonsynonymous SNV	0/1	1/1
3	chr10	89623901	rs2943772	PTEN	G	C	nonsynonymous SNV	/	1/1
4	chr20	56803624	rs146771462	ANKRD60	G	C	nonsynonymous SNV	1/1	0/0
5	chr3	75786555	rs141276988	ZNF717	-	TG	frameshift insertion	0/1	0/0
6	chr3	112253058	rs35560667	ATG3	-	A	frameshift insertion	0/1	1/1
7	chr1	1355796	rs145378993	ANKRD65	C	T	nonsynonymous SNV	0/1	0/0
8	chr10	69644589	rs548590752	SIRT1	C	T	nonsynonymous SNV	0/0	0/1
9	chr16	72042682	rs3213422	DHODH	A	C	nonsynonymous SNV	0/1	1/1
10	chr17	16256681	rs188652843	CENPV	G	A	nonsynonymous SNV	1/1	0/1
11	chr19	1000785	rs12986002	GRIN3B	C	T	nonsynonymous SNV	1/1	0/1
12	chr19	16436376	rs3745319	KLF2	G	A	nonsynonymous SNV	0/0	0/1
13	chr2	26407937	rs181971256	GAREM2	A	G	nonsynonymous SNV	0/0	0/1
14	chr2	100938226	rs74177694	LONRF2	G	C	nonsynonymous SNV	/	1/1
15	chr2	100938481	rs74177696	LONRF2	C	G	nonsynonymous SNV	0/0	0/1
16	chr2	128459214	rs10206957	SFT2D3	C	G	nonsynonymous SNV	1/1	0/1
17	chr2	202410300	rs10804117	C2CD6	A	T	nonsynonymous SNV	0/0	0/1
18	chr22	18923745	rs2008720	PRODH	G	T	nonsynonymous SNV	0/0	0/1
19	chr22	19137658	rs34341950	GSC2	G	A	nonsynonymous SNV	1/1	0/1
20	chr3	75788076	rs146581110	ZNF717	A	C	nonsynonymous SNV	/	1/1
21	chr3	75788085	rs201605431	ZNF717	A	G	nonsynonymous SNV	/	1/1
22	chr3	75788105	rs796745611	ZNF717	C	T	nonsynonymous SNV	/	1/1
23	chr3	75788109	rs796849627	ZNF717	G	A	nonsynonymous SNV	/	1/1
24	chr3	75788130	rs113708852	ZNF717	C	T	nonsynonymous SNV	/	1/1
25	chr3	75788137	rs199883677	ZNF717	C	T	nonsynonymous SNV	/	1/1
26	chr3	184017075	rs182086670	PSMD2	C	G	nonsynonymous SNV	0/1	1/1
27	chr4	80905990	rs12647691	ANTXR2	C	G	nonsynonymous SNV	1/1	0/1
28	chr6	42075069	rs55772414	C6orf132	A	G	nonsynonymous SNV	0/1	1/1
29	chr7	93540153	rs17243826	GNGT1	G	C	nonsynonymous SNV	1/1	0/1
30	chr8	8750243		MFHAS1	A	G	nonsynonymous SNV	0/0	0/1

0/1 denotes “heterozygous mutant”, 0/0 is for wild-type genotype and 1/1 is for homozygous mutants.

## Data Availability

All data generated or analyzed during this study are included in this article and its additional files. The WES datasets used and/or analyzed during this study are available from the corresponding author upon reasonable request.

## Supplementary Materials

The following supporting information can be downloaded at: https://www.mdpi.com/article/10.3390/genes13101689/s1, Table S1. GO of genes with sporadic mutations.
