# Supplementary material for: Whole-Exome Sequencing Reveals Pathogenic SIRT1 Variant in Brain Arteriovenous Malformation: A Case Report
Source: Genes (Basel). 2022 Sep 21;13(10):1689. doi: 10.3390/genes13101689 (PMC9601721; doi:10.3390/genes13101689)
Supplement: Supplementary file 1 [file genes-13-01689-s001.zip › genes-1910021-supplementary.pdf]

Table S1. GO of genes with sporadic mutations.

	Gene Set	Description	Size	Expect	Ratio	*p* Value	FDR
GO	GO:0010657	muscle cell apoptotic process	70	0.052490	57.154	0.000017142	0.022354 *
	GO:0044321	response to leptin	22	0.016497	121.23	0.00011808	0.076991
	GO:0033028	myeloid cell apoptotic process	33	0.024745	80.823	0.00026867	0.11678
	GO:0043491	protein kinase B signaling	232	0.17397	17.245	0.00060070	0.19583
	GO:0046677	response to antibiotic	316	0.23696	12.661	0.0014699	0.29757
	GO:0104004	cellular response to environmental stimulus	320	0.23996	12.502	0.0015240	0.29757
	GO:0070482	response to oxygen levels	337	0.25270	11.872	0.0017680	0.29757
	GO:0035690	cellular response to drug	349	0.26170	11.463	0.0019543	0.29757
	GO:0016605	PML body	99	0.074236	26.941	0.0024015	0.29757
	GO:0030099	myeloid cell differentiation	379	0.28420	10.556	0.0024728	0.29757
KEGG	hsa04068	FoxO signaling pathway	132	0.19970	20.030	0.000031031	0.010116 *
	hsa05031	Amphetamine addiction	68	0.10287	19.441	0.0044890	0.73171
	hsa04724	Glutamatergic synapse	114	0.17247	11.596	0.012220	1
	hsa04140	Autophagy	128	0.19365	10.328	0.015243	1
	hsa04371	Apelin signaling pathway	137	0.20726	9.6496	0.017341	1
	hsa05206	MicroRNAs in cancer	150	0.22693	8.8133	0.020580	1
	hsa04218	Cellular senescence	160	0.24206	8.2625	0.023233	1
	hsa05034	Alcoholism	180	0.27231	7.3444	0.028943	1
	hsa05167	Kaposi sarcoma-associated herpesvirus infection	186	0.28139	7.1075	0.030757	1
	hsa04744	Phototransduction	28	0.042360	23.607	0.041582	1
Disgenet	C0266011	Accessory nipple	11	0.0086421	231.42	0.000028205	0.10264
	C0020564	Hypertrophy	17	0.013356	149.75	0.000069598	0.12663
	C0027055	Myocardial Reperfusion Injury	37	0.029069	68.802	0.00033845	0.41054
	C0221352	Syndactyly of fingers	48	0.037711	53.035	0.00057102	0.41795
	C1859778	Postnatal growth retardation	57	0.044782	44.661	0.00080540	0.41795
	C0015695	Fatty Liver	60	0.047139	42.428	0.00089226	0.41795
	C0021655	Insulin Resistance	72	0.056567	35.356	0.0012831	0.41795
	C2051831	Pectus excavatum	100	0.078565	25.457	0.0024605	0.41795
	C0022658	Kidney Diseases	113	0.088778	22.528	0.0031312	0.41795
	C2936786	Aqueductal Stenosis	5	0.0039282	254.57	0.0039231	0.41795

* indicates $p \leq 0.05$ or "statistically significantly overrepresented".
